# Chromatic information processing in the first optic ganglion of the butterfly *Papilio xuthus*

**DOI:** 10.1007/s00359-019-01390-w

**Published:** 2019-12-14

**Authors:** Pei-Ju Chen, Gregor Belušič, Kentaro Arikawa

**Affiliations:** 1 Laboratory of Neuroethology, SOKENDAI, The Graduate University for Advanced Studies, Shonan Village, Hayama, 240-0193 Japan; 2 Department of Biology, Biotechnical Faculty, University of Ljubljana, Večna pot 111, 1000 Ljubljana, Slovenia

**Keywords:** Color vision, Interphotoreceptor synapses, Histamine-gated chloride channel, Spectral opponency, Lamina monopolar cell

## Abstract

The butterfly *Papilio xuthus* has acute tetrachromatic color vision. Its eyes are furnished with eight spectral classes of photoreceptors, situated in three types of ommatidia, randomly distributed in the retinal mosaic. Here, we investigated early chromatic information processing by recording spectral, angular, and polarization sensitivities of photoreceptors and lamina monopolar cells (LMCs). We identified three spectral classes of LMCs whose spectral sensitivities corresponded to weighted linear sums of the spectral sensitivities of the photoreceptors present in the three ommatidial types. In ~ 25% of the photoreceptor axons, the spectral sensitivities differed from those recorded at the photoreceptor cell bodies. These axons showed spectral opponency, most likely mediated by chloride ion currents through histaminergic interphotoreceptor synapses. The opponency was most prominent in the processes of the long visual fibers in the medulla. We recalculated the wavelength discrimination function using the noise-limited opponency model to reflect the new spectral sensitivity data and found that it matched well with the behaviorally determined function. Our results reveal opponency at the first stage of *Papilio*’s visual system, indicating that spectral information is preprocessed with signals from photoreceptors within each ommatidium in the lamina, before being conveyed downstream by the long visual fibers and the LMCs.

## Introduction

Color vision is a prominent sensory modality for most visual animals (Briscoe and Chittka 2001; Kelber et al. 2003). The process of color vision starts with light detection by photoreceptors with various spectral sensitivities. Insect photoreceptors depolarize in response to light and in turn release histamine, which hyperpolarizes postsynaptic neurons via histamine-gated chloride channels (Hardie 1987). These neuronal signals are further processed in the optic lobe before being sent to the central brain.

**Fig. 1:**
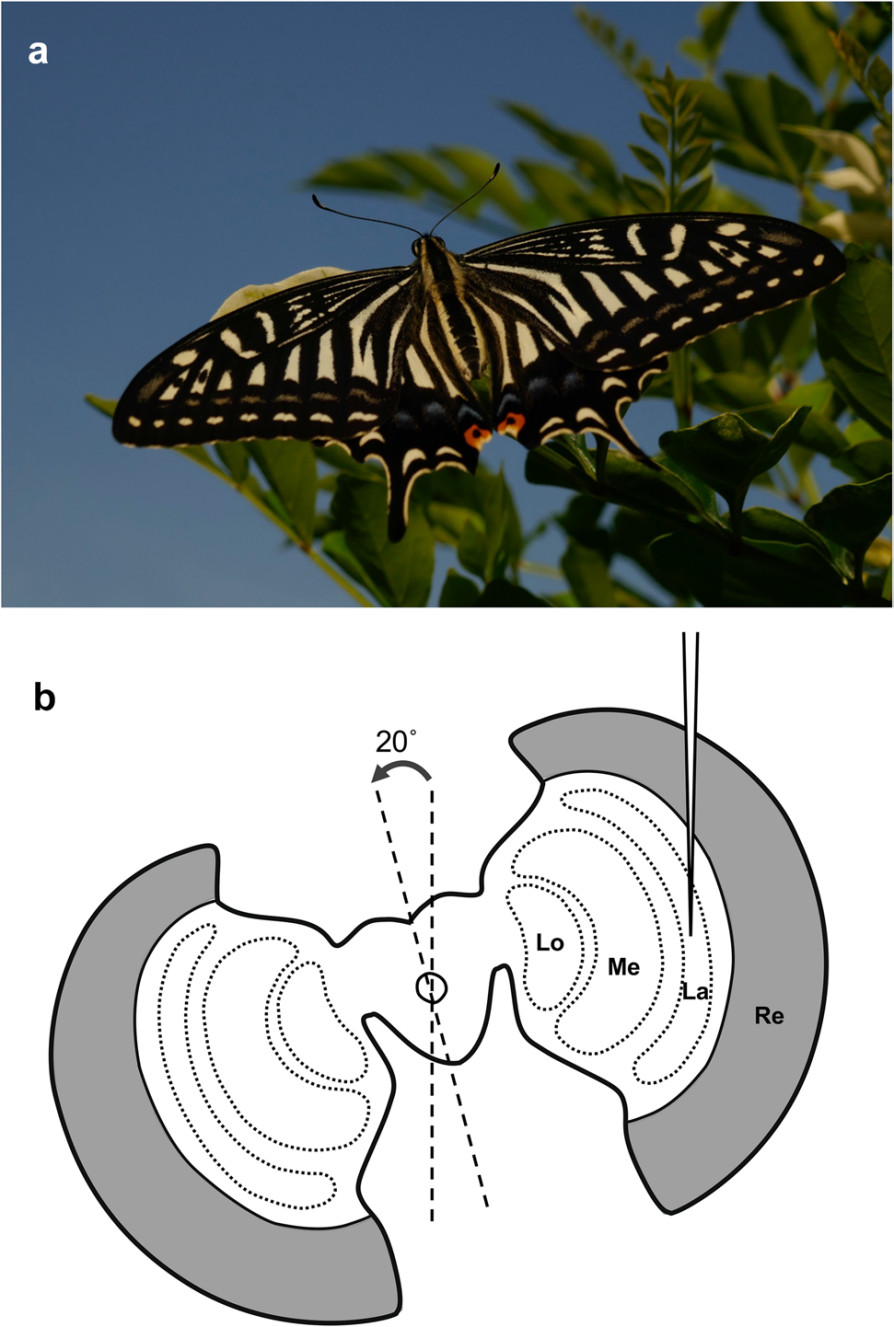
Preparation. **a** Adult *Papilio xuthus*. **b** Diagram of electrophysiological preparations. The head was tilted 20° to guide the electrode tip to the lamina (La) through the retina (Re). Lo, lobula; Me, medulla

Butterflies are renowned for their sophisticated color vision. While both the spectral organization of the compound eye and the behavioral features of color vision have been extensively studied (Kelber and Pfaff 1999; Kinoshita et al. 1999; Arikawa 2003; Kinoshita and Arikawa 2014), the neural mechanism underlying color vision remains unknown. The Japanese yellow swallowtail butterfly, *Papilio xuthus*, is a species whose color vision has been studied in detail (Fig. 1). These insects have a tetrachromatic system that enables them to perform the finest wavelength discrimination of any animal studied, including humans (Koshitaka et al. 2008; Thoen et al. 2014).

*Papilio* butterflies have six spectrally distinct classes of photoreceptors, which are embedded in the ommatidia, the units that comprise the compound eye. Each ommatidium contains nine photoreceptors (R1–R9) in one of three fixed combinations, making the eye a random mosaic of three types of spectrally heterogeneous ommatidia (Arikawa 2003). The photoreceptors in a single ommatidium taper toward the first optic ganglion, the lamina, where they are bundled together with four second-order neurons called lamina monopolar cells (LMCs) to form a lamina cartridge. The LMCs are postsynaptic to the photoreceptors in the cartridge with various spectral sensitivities (Takemura and Arikawa 2006). Interestingly, the photoreceptors are also postsynaptic to other photoreceptors: They are mutually connected via histamine-gated chloride channels in a configuration that depends on the ommatidial type (Takemura and Arikawa 2006; Akashi et al. 2018; Chen et al. 2019).

In the fruit fly *Drosophila melanogaster*, chromatic information processing with interphotoreceptor opponency starts in the second optic ganglion, the medulla. This is because the spectrally heterogeneous photoreceptors, R7 and R8, are long visual fibers (lvfs) whose axons terminate in the medulla without making any contacts with interneurons or other photoreceptors in the lamina. These photoreceptors mutually inhibit each other at their medulla terminals, producing spectral opponency: They respond positively to some wavelengths and negatively to others (Schnaitmann et al. 2018). On the other hand, the LMCs are spectrally homogeneous because they receive inputs only from the spectrally identical R1–R6 short visual fibers (svfs), which terminate in the lamina. LMCs are therefore mainly involved in non-chromatic visual processing (Borst 2014).

The situation is more complex in butterflies. In the *Papilio* eye, photoreceptors R1 and R2 are lvfs, which are either ultraviolet (UV), violet (V), or blue (B) sensitive. *Papilio*’s tetrachromacy is based on the UV, B, green (G), and red (R) receptors; G and R receptors are svfs. We have therefore hypothesized that chromatic information is transmitted to the medulla through at least two channels, one comprising the R1 and R2 lvfs, and the other comprising the LMCs receiving inputs from svfs. To address the possible roles of interphotoreceptor synapses and LMCs in chromatic information processing, we have undertaken an extensive analysis of the spectral properties of *Papilio* LMCs and photoreceptor axons in the lamina.

Here, we performed a series of electrophysiological analyses using glass microelectrodes inserted into the *Papilio* lamina. We first surveyed all recorded responses and categorized them into LMCs and photoreceptors. We then analyzed their response characteristics, focusing on their spectral properties. We found three spectral types of LMC, whose responses corresponded to the summed spectral sensitivities of the photoreceptors found in the three ommatidial types. Additionally, we observed that both LMCs and photoreceptors can be spectrally opponent. The spectral-opponent photoreceptors and the spectrally heterogeneous LMCs presumably provide the necessary inputs to the downstream chromatic circuitry that underlies the sophisticated color vision of *Papilio*.

## Materials and methods

### Animals

Both sexes of spring-form adult Japanese yellow swallowtail butterflies (*Papilio xuthus* Linnaeus) were obtained from a laboratory culture derived from eggs laid by wild females caught around the campus of Sokendai, Kanagawa, Japan. The hatched larvae were fed with fresh citrus leaves under a short light regime (light/dark = 10 h:14 h) at 25 °C. The pupae were stored at 4 °C for at least 3 months and then allowed to emerge at 25 °C.

### Light stimulation

The stimulus light was provided from a 500 W xenon arc lamp through a series of 23 narrow-band interference filters (IF) ranging from 300 to 740 nm at 20-nm intervals. The duration of each flash was fixed at 30 ms or 100 ms, with a 1-s interval between flashes. The light was guided through a quartz optical fiber providing a point light source about 1.6° in diameter from the animal’s perspective. Light intensity was controlled by a set of neutral density (ND) filters. The exit of the fiber was mounted on a perimeter device and positioned at the optical axis of the ommatidium containing the penetrated cell. The quantum flux of each monochromatic light was measured using a radiometer (model 470D; Sanso, Tokyo, Japan) and adjusted maximally to 5.0 × 10^11^ photons/cm^2^/sec at the corneal surface using an optical wedge.

We also used an LED (light-emitting diode) array device with 21 narrow-band LEDs with peak wavelengths of 365, 375, 390, 403, 422, 435, 451, 471, 495, 514, 525, 540, 560, 577, 590, 595, 620, 630, 657, 673, and 686 nm to produce light of arbitrary spectral content (Belušič et al. 2016). The quantum flux was controlled using 12-bit pulse width modulation at 1 kHz and a set of ND filters. The duration of each flash was fixed to 10 ms or 100 ms with 50-ms, 100-ms, or 150-ms intervals. Again, the light was aligned to the optical axis of the ommatidium in question. The angular size of the stimulating beam (min. ~ 2°, max ~ 20°) was adjusted with an aperture in the optical path.

### Intracellular electrophysiology

A butterfly with its wings and legs removed was mounted on a stage. To yield the longest possible electrode track in the lamina, the animal’s head was tilted approximately 20° to the right about the roll axis (Fig. 1). A chloridized silver wire was inserted into the stump of an antenna, to serve as the reference electrode. The left eye was positioned at the center of the perimeter device, which was set in a Faraday cage.

Microelectrodes were pulled from borosilicate glass capillaries (1 mm/0.5 mm outer/inner diameter) with a P-2000 laser micropipette puller (Sutter, Novato, CA, USA) and filled with 2 M potassium acetate. The resistance of the electrode was about 80–120 MΩ. To insert the glass microelectrode, a hole spanning about 20 ommatidia was made in the dorsal region of the eye. The microelectrode was advanced into the lamina through the retina, basement membrane, and the fenestrated layer.

The signal was amplified with a SEC-05X or SEC-10LX amplifier (Npi electronic, Tamm, Germany). Microelectrode resistance and capacitance were carefully compensated prior to recording and checked during the excursion through the tissue. Current injection was performed in discontinuous current clamp mode (dSECC) at the switching frequency max. 20 kHz. The signal was conditioned with a Cyber Amp 320 (Axon Instruments, Union City, CA, USA) and digitized with a Micro 1401 (CED, Cambridge, UK) analog–digital (A/D) converter. WinWCP (Strathclyde Electrophysiology Software, Version 4.0.5) and AcqKnowledge (BioPac Systems) packages were used for data acquisition and analysis.

Spectral responses were recorded by applying a series of equiquantal monochromatic flashes from short to long wavelength and then repeated in the reverse direction. Polarization responses were recorded at a given wavelength by rotating a polarization filter (OUV2500, Knight Optical, UK) in front of the exit pupil of the optical fiber: *ϕ* = 0° was defined as the vertical direction. To measure spatial responses, the motorized perimeter device swept vertically at 0.2° intervals through the center of the receptive field between dorsal (+ 5°) and ventral (− 5°) positions. The response–light intensity (*V*-log *I*) function was recorded over a 4 log unit intensity range at a given wavelength. The recorded responses were fitted to the Naka–Rushton function, *V*/*V*_max_ = *I*^*n*^/(*I*^*n*^ + *K*^*n*^), where *I* is the stimulus intensity, *V* is the response amplitude, *V*_max_ is the maximum response amplitude, *K* is the stimulus intensity eliciting 50% of *V*_max_, and *n* is the exponential slope. The *V*-log *I* function was used to convert the *V* values into photon numbers required to elicit the responses. The normalized reciprocal of the relative photon numbers then yielded the spectral, polarization, and angular sensitivities. Polarization sensitivities were fitted to the function $S\left( {\phi_{\text{stim}} } \right) = A\left[ {\cos \left( {\phi_{\text{stim}} - \phi_{\text{max} } } \right)} \right]^{2} + C$.

### Model calculation

To predict the wavelength discrimination ability, we modified the noise-limited color-opponent model (Vorobyev and Osorio 1998; Koshitaka et al. 2008). Here, we specifically focused on the interactions between lvfs and LMCs.

To calculate the derivatives of the spectral sensitivities *R*_*i*_(*λ*) of presynaptic cells *i*, the spectra were approximated as sums of Gaussian functions:

$$R_{i} \left( \lambda \right) = A_{i} \exp \left( {\frac{{ - \left( {\lambda - \lambda_{i}^{0} } \right)^{2} }}{{2\delta_{i}^{2} }}} \right) + B_{i} \exp \left( {\frac{{ - \left( {\lambda - \lambda_{i}^{1} } \right)^{2} }}{{2\sigma_{i}^{2} }}} \right) + C_{i} \exp \left( {\frac{{ - \left( {\lambda - \lambda_{i}^{2} } \right)^{2} }}{{2\rho_{i}^{2} }}} \right),$$

where $A_{i}$, $B_{i}$, $C_{i}$, $\lambda_{i}^{0}$, $\delta_{i}$, $\lambda_{i}^{1}$,$\sigma_{i}$, $\lambda_{i}^{2}$, and $\rho_{i}$ are parameters (see Table 1) whose values were adjusted to provide a best fit to the observed spectral sensitivities of photoreceptors and LMCs. We ignore the distinction between narrow-band blue (nB) and wideband blue (wB) receptors, fitting a single blue lvf function to the average of these two sensitivity spectra.

**Table 1:** Parameters of the Gaussian function to approximate spectral sensitivities of long visual fibers (lvfs) and lamina monopolar cells (LMCs, see “Materials and methods” and Koshitaka et al. 2008)

	*A*	*λ*^0^	*δ*	*B*	*λ*^1^	*σ*	*C*	*λ*^2^	*ρ*
UV	1	365	32	0	–	–	0	–	–
V	1	403	18	0	–	–	0	–	–
B	1	455	27	0.46	372	40	0	–	–
LMC I	1	610	40	0.85	500	40	0.75	370	50
LMC II	1	560	60	0.95	460	50	0.4	600	50
LMC III	1	510	55	0.95	360	32	0	–	–

The relative noise level is inversely proportional to the number of cells of a particular type, $\omega_{i} = v_{i} /\sqrt {\eta_{i} }$, where *v*_*i*_ is the noise level of a cell and *η*_*i*_ is the number of cells of type *i*. We assume that different cells have similar levels of noise and set the noise of type I LMCs to 0.05, i.e., $\omega_{i} = \sqrt {\eta_{I} /\eta_{i} }$. The number of cells of each type is based on the putative composition of each medulla column, according to the type of ommatidium to which it corresponds: one UV lvf, one blue lvf, and four type I LMCs for type I; two violet lvfs and four type II LMCs for type II; and two blue lvfs and four type III LMCs for type III. Details of the cell types are described below.

## Results

The electrode inserted into the retina typically reached the fenestrated layer, a trachea-rich region between the retina and the lamina, after advancing 400–500 μm. It would then pass into the lamina, where among the successively impaled cells we encountered types with negative- and/or positive-going responses to light pulses. We first analyzed the general properties of these responses to infer whether they originated from lamina monopolar cells (LMCs) or photoreceptors.

### Identification of lamina monopolar cell (LMC) responses

**Fig. 2:**
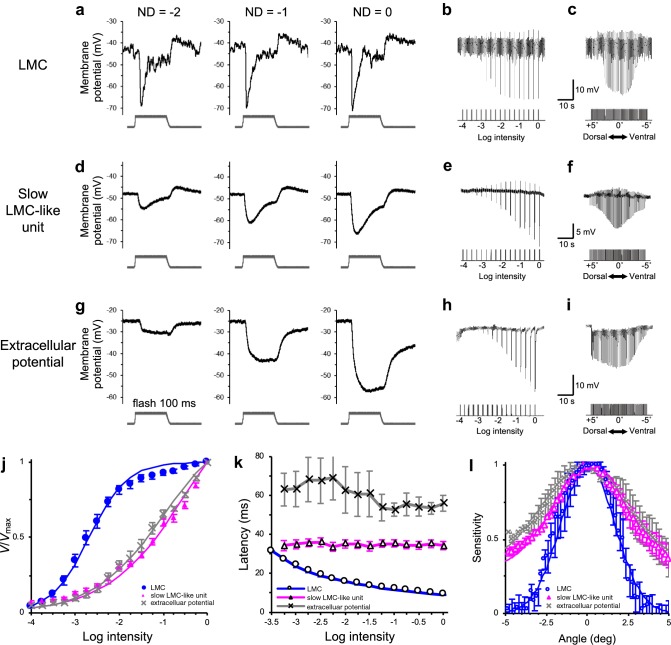
Light responses of hyperpolarizing units in the *Papilio* lamina. **a–i** Membrane potential traces of a typical LMC (**a–c**), a slow LMC-like unit (**d–f**) and extracellular potential (**g–i**). **a**, **d**, **g** Waveforms recorded at three light intensities, increasing (from left to right) in intervals of 1 log unit. **b**, **e**, **h** Responses to 100 ms pulses of 540 nm light of increasing intensity, in 0.25 log unit steps. **c**, **f** and **i** Spatial responses measured with angular steps of 0.2° over 10° (dorsal (+ 5°) to ventral (− 5°)) with 30 ms pulses at 540 nm. **j** Normalized *V*/*V*_max_ curves of hyperpolarizing responses of a typical LMC (*N *= 31), slow LMC-like units (*N *= 13), and extracellular potential (*N *= 5) to different light intensities. The *lines* are the best fits to the Naka–Rushton function: For typical LMCs, the exponential slope *n* is 1.02 and the stimulus intensity eliciting 50% *V*_max_ (log*K*) is − 2.68; for slow LMC-like units *n* = 0.47 and log*K* = − 0.4; and for extracellular potential *n* = 0.54 and log*K* = − 1.01. **k** Latencies (i.e., time taken for the membrane potential to reach its peak) of hyperpolarizing on-transients under different light intensities for typical LMCs (*N *= 31), slow LMC-like units (*N *= 13), and extracellular potentials (*N* = 5). **l** Angular sensitivities of typical LMCs (*N *= 6), slow LMC-like units (*N *= 6), and extracellular potentials (*N *= 2). The *lines* are the best fits to Gaussian functions

We have encountered three types of negative-going responses that could potentially be assigned to LMCs (Fig. 2). We ultimately accepted only the first type as true instances of LMCs (Fig. 2a–c) for further analyses, excluding the second (Fig. 2d–f) and third (Fig. 2g–i) types, which we describe as slow LMC-like units and extracellular potentials, respectively. The “LMC” type exhibits a noisy membrane potential, and its response to light consists of an initial fast negative on-transient, then a plateau, and finally a positive off-transient (Fig. 2a). These characteristics clearly differed in the other types of units, which also responded negatively to light, but lacked the plateau and/or positive off-transient (Fig. 2g). The “slow LMC-like” units (Fig. 2d–f) were often encountered in the distal lamina immediately after the electrode had passed the fenestrated layer.

We first compared the slopes of these recordings’ intensity–response curves (the exponential slope parameter *n* being inversely proportional to the dynamic operating range of the cell, Fig. 2j), their latencies (the time from the light onset to the negative peak, Fig. 2k), and their acceptance angles (half width of the angular sensitivity function, Fig. 2l). The *n* (exponential slope) value of the *V*-*log I* function is the largest in the “LMC” type, which means their dynamic range is the narrowest. This is a distinctive characteristic of previously reported insect LMCs (Laughlin 1973; de Souza et al. 1992). The latency shortens as the intensity of the stimulus increases in the “LMC” type. The acceptance angles of the three responses types are 3.9°, 7.3°, and 8.6°, respectively (Fig. 2l). Even the value recorded in the “LMC” type, 3.9°, is considerably larger than the acceptance angle of a single ommatidium measured in the central part of the retina, 1.9° (Takeuchi et al. 2006). The LMCs may therefore receive inputs from more than one ommatidium, but this remains an unconfirmed hypothesis at present.

The on-transient and the sustained component of an “LMC” response to light are generated mainly due to an increase in the membrane conductance via the opening of histamine-gated chloride channels (Hardie 1989). Thus, we carried out current clamp analyses to confirm the reversal potential of the hyperpolarizing responses.

**Fig. 3:**
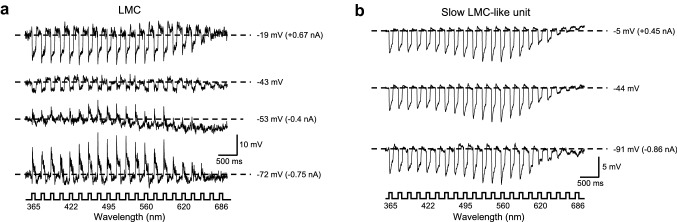
Responses of an LMC (**a**) and a slow LMC-like unit (**b**) to current injection. The cells were stimulated with an isoquantal spectral sequence of 100 ms pulses and with a positive (top trace), zero (middle trace), and negative (bottom trace) current injection. The numbers on the right indicate the membrane potential with the injected current in brackets

The response amplitude and polarity of the “LMC” could be altered by the current injection (Fig. 3a). The on-transients and the plateau were virtually eliminated when the membrane potential was changed from its resting value (− 43 mV) to − 53 mV; partial depolarization (from − 43 to − 19 mV) resulted in an increase in the amplitude of the light responses; finally, hyperpolarization to − 72 mV resulted in the reversal of the polarity of light responses. This indicates that the light responses of the “LMC” units were caused by an ionic current with reversal potential *E *≈ − 53 mV, possibly close to the Cl^−^ equilibrium potential. In contrast, current injections in the “slow LMC-like” units failed to produce any clear changes in responses to light flashes (Fig. 3b). We assume that these recordings were obtained from the LMC somata, which are connected to the cells’ synaptic region via a long process (Hamanaka et al. 2013). The process is very thin and therefore has high resistance, acting as a low-pass filter for the responses to receptor input and preventing any manipulation of these responses by the current injection. Drawing these lines of evidence together, we have concluded that the first “LMC”-type responses were indeed recorded from single LMCs in the main part of the lamina.

In our previous study, we reported that depolarizing off-spikes could be recorded in some *Papilio* LMCs (Rusanen et al. 2018). However, we found that the off-spike generation was an unstable trait, presumably related to the physiological properties of individual LMCs, which seem to depend on the age of the individual (Table 2). We therefore did not examine the off-spiking property in this study.

**Table 2:** Recording numbers of large monopolar cell types, spectral classes, and spiking properties

LMC type	Opponency	Spectral class		Non-spiking	Spiking
LMC *N *= 169 (84.2%)	Non-opponent *N *= 130 (76.9%)	I	*N *= 57 (43.8%)	*N *= 46	*N *= 11
		II	*N *= 17 (13.1%)	*N *= 14	*N *= 3
		III	*N *= 55 (42.3%)	*N *= 54	*N *= 1
		Others	*N *= 1 (0.8%)	*N *= 1	*N *= 0
	Opponent *N *= 39 (23.1%)	SW	*N *= 7 (17.9%)	*N *= 6	*N *= 1
		MW	*N *= 4 (10.3%)	*N *= 3	*N *= 1
		LW	*N *= 23 (59.0%)	*N *= 10	*N *= 8
		Others	*N *= 5 (12.8%)	*N *= 5	*N *= 0
Slow LMC-like unit *N *= 27 (13.8%)	Non-opponent *N *= 27 (100%)			*N *= 27	*N *= 0

### Spectral properties of lamina monopolar cells (LMCs)

We analyzed 169 LMC recordings (Table 2), all of which exhibited responses to a broad spectrum of light. We first divided these into two types: non-spectral-opponent, which did not depolarize at any wavelength, and spectral-opponent, which hyperpolarized at some wavelengths and depolarized at others. The LMCs were then grouped into different spectral classes according to their spectral sensitivities (Table 2).

**Fig. 4:**
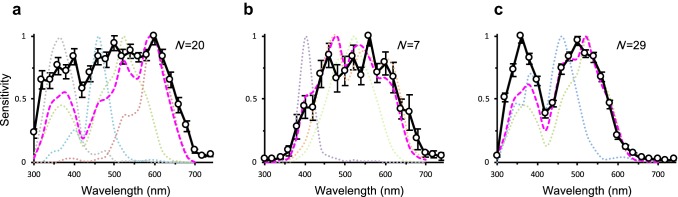
Spectral sensitivities of non-opponent LMCs. Average spectral sensitivities (mean ± SEM; thick solid lines) of three spectral classes correspond to the weighted linear sums (magenta dashed lines) of receptor sensitivities (thin dotted lines) of the three ommatidial types. **a** Cells with high UV and red sensitivity in type I ommatidia. **b** Cells with high red but low UV sensitivity in type II ommatidia. **c** Cells with high UV but low red sensitivity in type III ommatidia. *N* sample number. Error bars indicate SEM

About 77% of LMCs were of the non-opponent type. Spectral sensitivities of them are variable, particularly in the UV (300–360 nm) and R (600–700 nm) wavelength regions. We subdivided the non-opponent LMCs further into three spectral classes—I, II, and III—based on their sensitivity to these two wavelength regions (Fig. 4). Class I includes LMCs with high sensitivity to both UV and R (Fig. 4a): The sensitivities at 360 nm and 620 nm were over 60% of the maximum sensitivity. Class II includes LMCs with low UV and high R sensitivity (Fig. 4b): The response amplitude at 360 nm was less than 30%, and at 620 nm it was over 60% of the maximum sensitivity. Class III includes LMCs with high UV and low R sensitivities (Fig. 4c): Their sensitivity at 360 nm was more than 70%, and at 620 nm it was less than 30% relative to the maximum sensitivity. Thick solid lines in Fig. 4 represent average spectral sensitivities of each class from 56 LMCs where V-log I functions were successfully recorded.

The compound eye of *P*. *xuthus* is composed of three spectrally distinct types of ommatidia. Type I contains UV, nB, dG, and R receptors, while type II contains V, sG, and BB receptors. Type III has only wB and G receptors (Koshitaka et al. 2008). The LMC spectral sensitivities appear to be explained if we assume that each LMC receives input from all photoreceptors in its own cartridge. Therefore, we compared the LMC spectral sensitivities with a linear sum of receptor spectral sensitivities *S*(*λ*), each multiplied to reflect the number of receptors $n$ of that type in the ommatidium:

$$S\left( \lambda \right) = \mathop \sum \limits_{i = 1}^{8} n_{i} R_{i} \left( \lambda \right),$$

where *R*_*i*_(*λ*) is the spectral sensitivity of photoreceptor *i* (R1–R8: the ninth, basal R9 photoreceptors are omitted here because their spectral properties are not well established). The magenta dashed lines in Fig. 4 show the normalized weighted linear sum of photoreceptor spectral sensitivities in each ommatidial type (colored dotted lines in Fig. 4). The spectral sensitivities of the three classes of non-opponent LMCs indeed resemble the sums of receptor sensitivities in the three ommatidial types, suggesting that the majority of LMCs receive inputs from all spectral receptor types within the same cartridge. Accordingly, we describe the non-opponent LMCs with high UV/high R sensitivity as type I LMCs (Fig. 4a), low UV/high R sensitivity as type II (Fig. 4b), and high UV/low R sensitivity as type III (Fig. 4c).

**Fig. 5:**
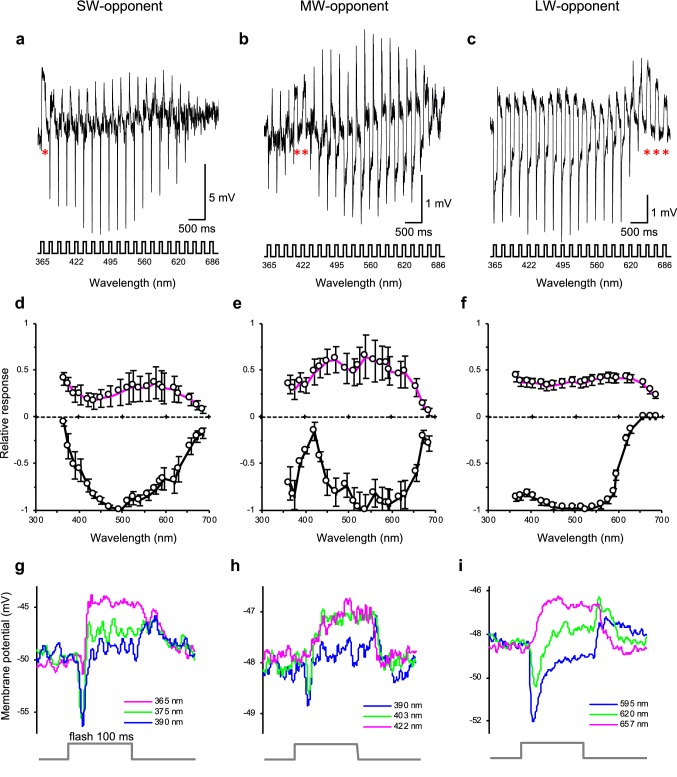
Spectral responses of spectral-opponent LMCs. **a–c** Membrane potential traces of a SW-opponent LMC (**a**), a MW-opponent LMC (**b**), and a LW-opponent LMC (**c**) upon isoquantal spectral stimulation with 100 ms pulses from the LED array. *Asterisks* indicate the depolarizing spectral-opponent responses. **d**–**f** Averaged spectral response curves (mean ± SEM) of short wavelength (SW)-opponent LMCs (**d**; *N *= 7), middle wavelength (MW)-opponent LMCs (**e**; *N *= 4), and long wavelength (LW)-opponent LMCs (**f**; *N *= 23). The black and magenta curves in **d**–**f** indicate averaged amplitudes of hyperpolarizing and depolarizing responses, respectively. **g**–**i** Expanded timescale showing temporal details of the responses from **a**–**c** at the wavelengths producing hyperpolarization (blue), mixed potential changes (green), and depolarization (magenta)

About 23% of recorded LMCs were spectral-opponent. We grouped them according to the wavelength region where they depolarize: UV- or SW-opponent, blue-violet- or MW-opponent, and R- or LW-opponent (Fig. 5). The SW-opponent LMCs showed peak hyperpolarization at 480–540 nm and depolarized below 380 nm (Fig. 5a, asterisk). The MW-opponent LMCs hyperpolarized from UV to R wavelengths, except for a narrow wavelength region (400–450 nm) where they depolarized (Fig. 5b, asterisks). The LW-opponent LMCs depolarized in the wavelength region above 630 nm (Fig. 5c, asterisks).

The depolarization was observed at the wavelengths where hyperpolarization was absent (Fig. 5d–f). The onset of depolarization occurred slightly later than the hyperpolarizing on-transients (Fig. 5g–i). The depolarization could be clearly distinguished from the depolarizing off-transients because it appeared within the duration of the light stimulus.

### Identification of photoreceptor responses in the lamina

We recorded a number of photoreceptor-like responses from the lamina. About a quarter of them responded both positively and negatively, depending on the wavelength. These units closely resemble the spectral-opponent photoreceptors reported in the retinas of a few butterfly species (Matić 1983; Chen et al. 2013).

**Fig. 6:**
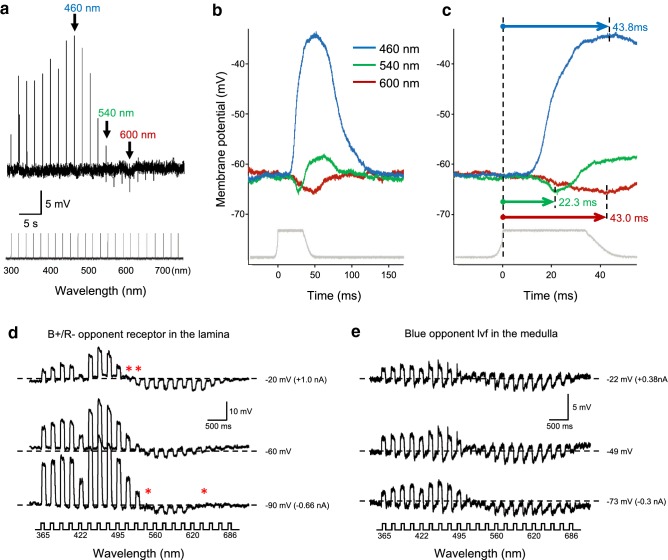
Response profiles of blue-sensitive opponent photoreceptors. **a–c** A B +/R− receptor recorded in the lamina. **a** Responses to 30 ms pulses of isoquantal monochromatic light from 300 to 740 nm, at 20-nm intervals. **b** Waveforms of the recording in **a** at 460 nm, 540 nm, and 600 nm. **c** As **b**, but with an expanded time axis. **d**, **e** Current injection experiments in a B +/R− receptor in the lamina (**d**) and a blue opponent lvf in the medulla (**e**). The cells were stimulated with 100 ms isoquantal spectral pulses of 21 wavelengths with positive (top), zero (middle), and negative (bottom) current injection. The numbers on the right are the membrane potential with the injected current in brackets. Asterisks in **d** indicate the response polarity reversal during the current injection

Figure 6 shows the responses of a typical blue-positive/red-negative (B +/R−) opponent unit. This unit displays a positive (depolarizing) peak at 460 nm and a negative (hyperpolarizing) peak at 600 nm. At 540 nm, the response is composed of an early hyperpolarization that is followed by a depolarization (Fig. 6a–c). Figure 6d shows the results of current injection experiments in a B +/R− unit. When the membrane potential was hyperpolarized to about 30 mV below the resting potential, some hyperpolarizing light responses were eliminated or even reversed at certain wavelengths, suggesting that the hyperpolarizations were due to antagonistic interactions through synaptic connections.

**Fig. 7:**
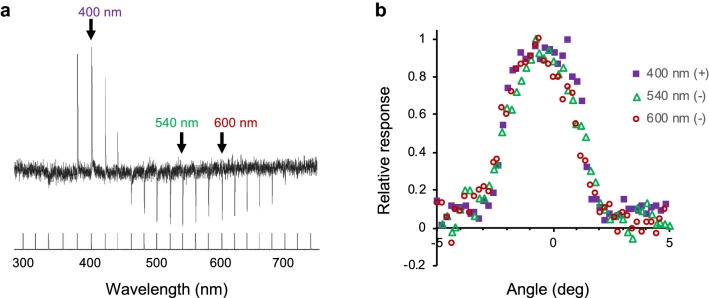
Angular responses of a V +/G− receptor. **a** Responses to 30 ms isoquantal monochromatic pulses from 300 to 740 nm. **b** Relative angular responses at 400, 540, and 600 nm with angular steps of 0.2° over 10°. The acceptance angle is independent of response polarity

We investigated the spatial origin of these antagonistic interactions by measuring the cell’s response as a function of the angular position of the light source. Figure 7 shows the angular responses measured at three different wavelengths in a V +/G− unit. The angular response profile was independent of wavelength, suggesting that the putative antagonistic interaction takes place within a single ommatidium.

**Fig. 8:**
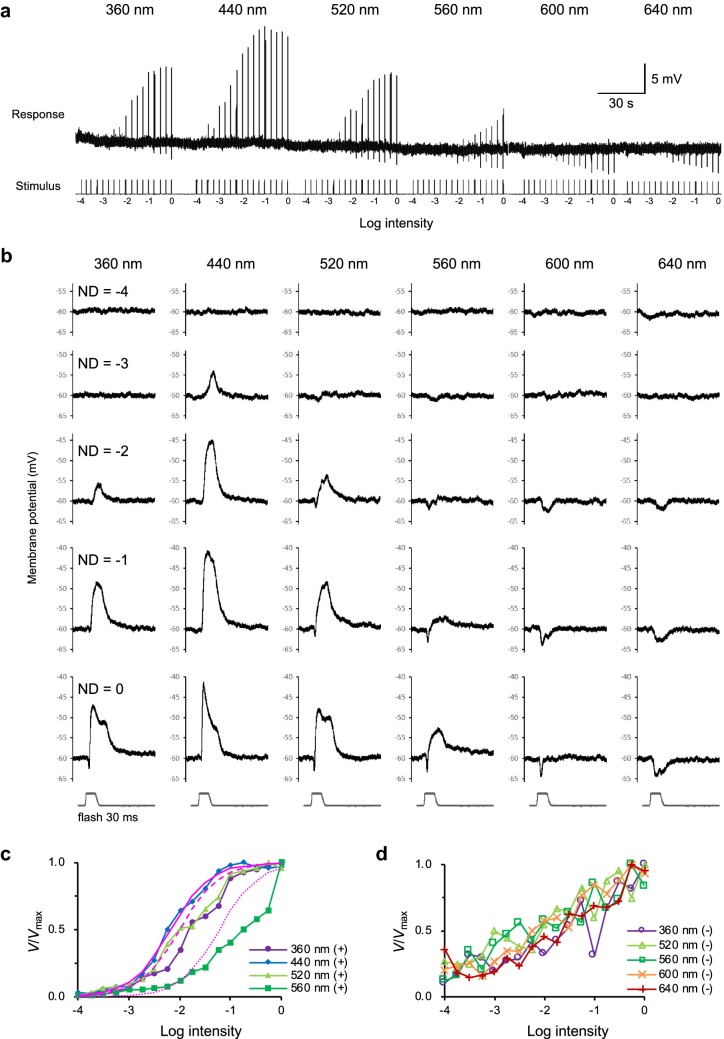
Spectral and intensity responses of a B +/R− receptor. **a** Responses to monochromatic light of six wavelengths at 0.25 log unit intensity steps. **b** Expanded traces from **a** at five intensities. **c** Four *V*/*V*_max_ curves of depolarizing responses shown in **a** measured at 440, 520, 360, and 560 nm. The smooth solid line is the Naka–Rushton function fitted to the responses to 440 nm. Dashed and dotted lines are the same curve shifted along the intensity axis to align with the responses to low intensity 360 nm and 560 nm, respectively. **d** Five *V*/*V*_max_ curves of hyperpolarizing responses from the recording in **a** measured at 360, 520, 560, 600, and 640 nm

To test whether the antagonistic responses originated from distinct photoreceptors with different intensity–response characteristics, we recorded responses of the spectral-opponent photoreceptors using a series of graded pulses at different wavelengths. Figure 8 shows the analysis of the wavelength–intensity–response relationship in a B +/R− unit at six wavelengths and five intensities. The unit responded purely positively at 440 nm, purely negatively at 600 nm and 640 nm, and showed mixed responses at other wavelengths (Fig. 8a, b). The 640 nm stimuli elicited slow negative responses, while at 600 nm, a fast onset negative response was observed at high intensities (Fig. 8b). This response appears to be a combination of the fast, strong hyperpolarization from red receptors and the slower, weaker endogenous depolarization from the blue receptor itself.

In the absence of synaptic input, photoreceptor responses to flashes of graded intensities at different wavelengths should yield a set of parallel *V*-log *I* functions offset along the abscissa, due to the principle of univariance (Arikawa et al. 2003). However, the *V*-log *I* functions of the B +/R− unit for different wavelengths do not exhibit this property, likely due to antagonistic influences from different photoreceptors with different intensity–response relations, via synapses with unknown gain. The solid line in Fig. 8c is the Naka–Rushton function, *V*/*V*_max_ = *I*^*n*^/(*I*^*n*^ + *K*^*n*^), for the positive responses fitted to the measurement at 440 nm. We took the same function (i.e., with parameters obtained at 440 nm) and transposed it to the right, aligning it to the low light intensity responses of the 520 nm and 560 nm series, and plotted these with dashed and dotted lines, respectively. The transposed 440 nm function deviates markedly from the actual measurements at intermediate intensities for 520 and 560 nm light, suggesting inhibition from long-wavelength-sensitive photoreceptors. These discrepancies between the predicted function and the data were diminished at the highest intensity, where the responses saturated. By contrast, the normalized *V*/*V*_max_ curves of the negative response components of the same B +/R− unit did not differ significantly among five wavelengths (Fig. 8d). This suggests that the recording was from a blue receptor’s axon, where the effects of inhibitory inputs from other photoreceptors were evident at all measured wavelengths except for its own sensitivity peak at 440 nm.

**Fig. 9:**
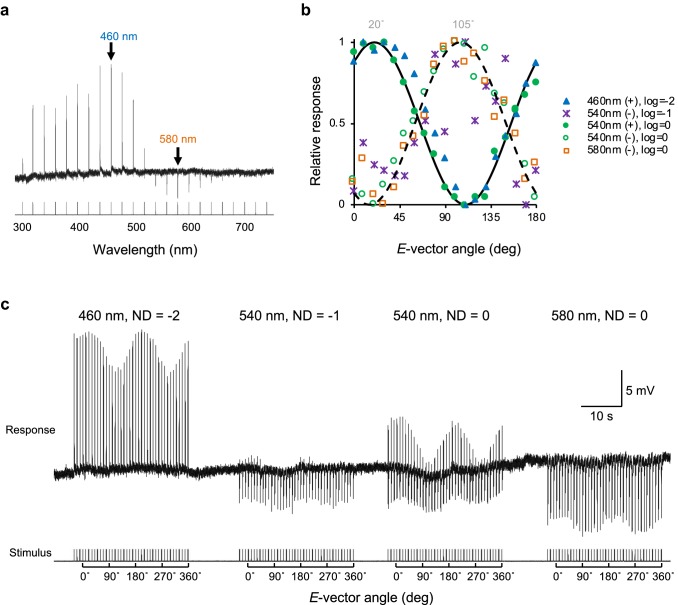
Polarization responses of a B +/R− receptor. **a** Responses to 30 ms pulses of isoquantal monochromatic light from 300 to 740 nm with 20-nm interval. **b** Normalized polarization response curves based on the responses shown in **c**. Solid and dashed lines are the best-fit cos^2^ curves for depolarizing (+) and hyperpolarizing (−) responses, respectively. (**c**) Voltage response trace of the cell to 30 ms pulses of light through a rotating polarizer at various wavelengths and intensities. Note that the response set “540 nm, ND = 0” corresponds to two plots (filled and open green circles) in **b**

All photoreceptors in *Papilio* have pronounced polarization sensitivity with phase-shifted sinusoidal sensitivity functions that correspond to the different orientations of the microvilli in a single rhabdom (Bandai et al. 1992). Thus, we expected to find phase-shifted polarization sensitivity functions for depolarizing and hyperpolarizing responses, because we hypothesize that these originate from different photoreceptors. To test this, we measured the polarization sensitivities of a B +/R− unit at different wavelengths (Fig. 9). The unit exhibited depolarizing responses at the blue receptor’s peak wavelength (460 nm) and hyperpolarizing responses at 540 nm and 580 nm. For a high-intensity series at 540 nm, the cell produced biphasic mixed responses, i.e., depolarizing and hyperpolarizing responses to the same flash (Fig. 9c), which are separately plotted in Fig. 9b. The depolarizing responses to flashes presented through a rotating polarizer followed the same cos^2^ function at all wavelengths (Fig. 9b); this was also the case for the hyperpolarizing responses. However, the depolarizing and hyperpolarizing responses were offset by ~ 90°, suggesting that the depolarizing response originated from a blue receptor, positioned at ~ 20° with respect to the polarizer, while the antagonistic inputs could be attributed to the R3–R4 green receptors, which have microvilli orthogonal to those of the blue receptor (Bandai et al. 1992). This arrangement can be described as polarization opponency. The 20° angular offset can be explained by the 20° tilt of our preparation for the recording (see “Materials and methods”).

### Classes of spectral-opponent photoreceptors

**Fig. 10:**
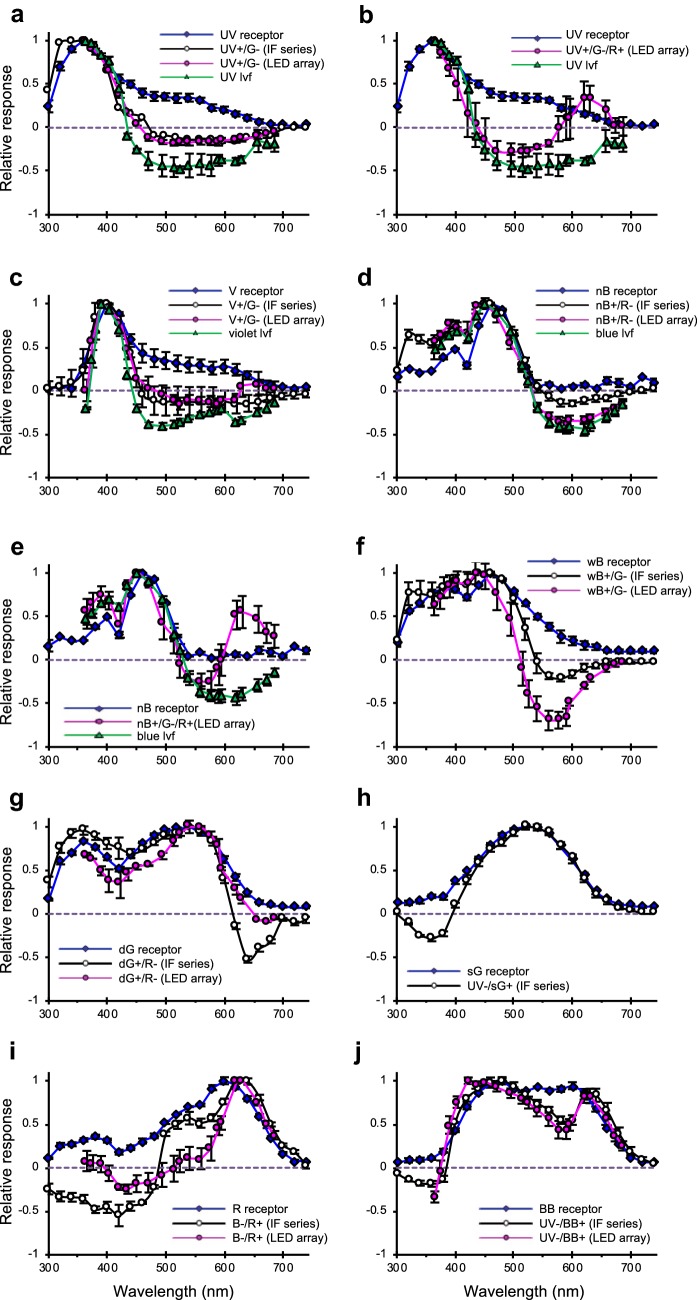
Dependence of photoreceptor spectral response on the depth of the recording site. Averaged spectral response curves (mean ± SEM) of non-opponent photoreceptors recorded by the IF series in the retina (blue lines with diamonds) as well as spectral-opponent photoreceptors recorded using the IF series (black lines with open circles) and the LED array in the lamina (magenta lines with solid circles) and in the medulla (green lines with triangles). **a** UV receptor (*N *= 11), UV +/G− receptor (*N *= 1 by the IF series*/N *= 6 by the LDE array), and UV lvf (*N *= 2). **b** UV receptor (*N *= 11), UV +/G−/R + receptor (*N *= 0/2), and UV lvf (*N *= 2). **c** V receptor (*N *= 6), V +/G− receptor (*N *= 3/4), and V lvf (*N *= 3). **d** nB receptor (*N *= 3), nB +/R− receptor (*N *= 4/13), and B lvf (*N *= 6). **e** nB receptor (*N *= 3), nB +/R− receptor (*N *= 0/4), and B lvf (*N *= 6). **f** wB receptor (*N *= 8) and wB +/G− receptor (*N *= 2/5). **g** dG receptor (*N *= 34) and dG +/R− receptor (*N *= 1/2). **h** sG receptor (*N *= 7) and UV−/sG + receptor (*N *= 1/0). **i** R receptor (*N *= 11) and B−/R + receptor (*N *= 1/5). **j** BB receptor (*N *= 8) and UV−/BB + receptor (*N *= 6/8). *BB* broadband, *dG* dual-peaked green, *nB* narrow blue, *R* red, *sG* single-peaked green, *UV* ultraviolet, *V* violet, *wB* wide blue

We first categorized the spectral-opponent photoreceptor units into six classes based on the wavelength of their peak positive (depolarizing) response: UV, violet (V), blue (B), green (G), red (R), and broadband (BB), which correspond to the spectral classes of photoreceptors previously identified in the retina (Arikawa 2003). We further divided each spectral class into subclasses according to the wavelengths at which they hyperpolarized (Table 3, Fig. 10). These subclasses comprised two UV-positive (UV +/G− and UV +/G−/R +), three B-positive (nB +/R− and nB +/G−/R + derived from narrow-band blue (nB) receptors in type I ommatidia, and wB +/G− derived from wideband blue (wB) receptors in type III ommatidia), and two G-sensitive (dG +/R− derived from dual-peaked green (dG) receptors and UV−/sG + derived from single-peaked green receptors).

**Table 3:** Recording numbers of spectral-opponent photoreceptors in the lamina

Spectral class		Ratio	Light stimulation		Graphs in Fig. 10
			LED array	IF series	
UV	UV +/G−	*N *= 7 (10.3%)	*N *= 6	*N *= 1	a
	UV +/G−/R+	*N *= 2 (2.9%)	*N *= 2	*N *= 0	b
Violet	V +/G−	*N *= 7 (10.3%)	*N *= 4	*N *= 3	c
Blue	nB +/R−	*N *= 17 (25.0%)	*N *= 13	*N *= 4	d
	nB +/G−/R+	*N *= 4 (5.9%)	*N *= 4	*N *= 0	e
	wB +/G−	*N *= 7 (10.3%)	*N *= 5	*N *= 2	f
Green	dG +/R−	*N *= 3 (4.4%)	*N *= 2	*N *= 1	g
	UV−/sG+	*N *= 1 (1.5%)	*N *= 0	*N *= 1	h
Red	B−/R+	*N *= 6 (8.8%)	*N *= 5	*N *= 1	i
Broadband	UV−/BB+	*N *= 14 (20.6%)	*N *= 8	*N *= 6	j

We also recorded units in the medulla, which we identified as receptor axons based on their sustained graded responses to light. Recording in the medulla was characterized by a deep electrode tip position and numerous spiking units penetrated during the excursion (in the lamina, spiking units were rarely encountered). The spectral-opponent units in the medulla were most likely the terminals of R1 and R2 long visual fibers (lvfs). These can be UV (Fig. 10a, b), V (Fig. 10c), or B (Fig. 10d, e) class. All of them depolarized at short wavelengths and hyperpolarized at long wavelengths.

The lvfs recorded in the medulla could be distinguished from the spectral-opponent photoreceptors in the lamina. First, the amplitude of hyperpolarization was greater in the medulla (about 50% relative to depolarization) than in the lamina (about 10–30% relative to depolarization). This is probably due to the accumulation of inhibitory inputs throughout the length of the lamina cartridge. Second, the maximal amplitude of depolarizing responses recorded in the medulla was smaller than in the lamina, due to the passive attenuation of graded signals propagated from the photoreceptor cell body through the long axon. The depolarization of lvfs saturated at lower light intensity (data not shown). Third, we could never successfully clamp any light-induced hyperpolarization by the current injection (Fig. 6e), indicating that the presumptive antagonistic synapses were too far from the recording site.

We compared the spectral responses of photoreceptors recorded in the lamina and medulla to those recorded in the retina (Fig. 10). The spectral-opponent units exhibited narrower spectra (e.g., wB +/G− and B−/R +). In the short- and mid-wavelength receptors, the tail extending into the long-wavelength region is absent (e.g., UV +/G−, V +/G−, and wB +/G−). The narrowing of the spectral sensitivity is much less pronounced in the green receptors, which is consistent with the observation that R3 and R4 have fewer interphotoreceptor synapses (Takemura and Arikawa 2006). In the case of the broadband (BB) receptor, identified in its spectral-opponent version as the UV-/BB + receptor, the sensitivity is suppressed in the UV range and in a narrow spectral band from 520 to 600 nm.

## Discussion

**Fig. 11:**
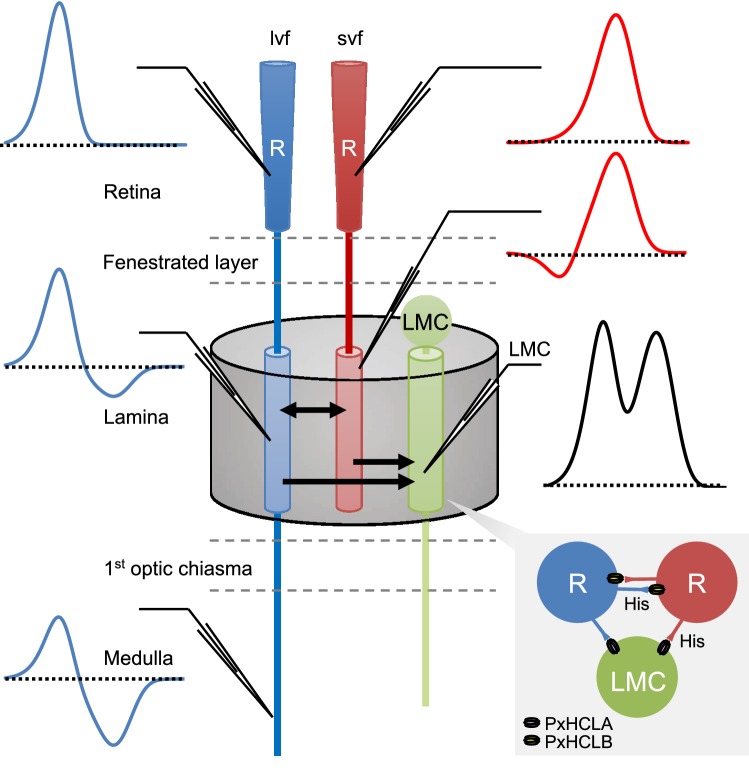
Summary diagram of a lamina cartridge showing possible neuronal circuits underlying the observed spectral opponency. Photoreceptors relay signals to LMCs via the PxHCLA (*Papilio xuthus* histamine-gated chloride channel A). Interphotoreceptor synapses employ PxHCLB (Chen et al. 2019). Photoreceptors depolarize upon light stimulation exhibiting distinct spectral sensitivities in the retina. Photoreceptors with distinct spectral sensitivities mutually inhibit in the lamina creating spectral opponency. LMCs receive inputs from all photoreceptors in the cartridge, and therefore exhibit broad spectral sensitivity. Long visual fibers (UV, V, and B receptors) feed the final, accumulated opponent responses to medulla neurons. lvf: long visual fiber. svf: short visual fiber. His: histamine

Our study has revealed complex spectral opponency at the first stages of visual processing in a butterfly. The neuronal circuit underlying the spectral opponency is summarized in Fig. 11, which will be referred in the discussion below in places. The LMCs constitute three spectrally distinct classes of visual interneurons, receiving inputs from all photoreceptors within their own ommatidium, including the lvfs. The LMCs’ spectral sensitivities are therefore broad, spanning 300–700 nm, but heterogeneous between ommatidial types (Fig. 11). In the photoreceptors, opponent processing can be detected only ~ 200 µm proximal from the usual recording site in the retina. Interphotoreceptor synapses produce a variety of spectral-opponent responses at the photoreceptor terminals in the lamina and in the medulla. In flies, the functionally characterized LMCs feed information mainly to the motion detection system (Borst et al. 2010; Joesch et al. 2010). This is probably the case also in *Papilio*, where motion detection can be triggered by purely chromatic contrast (Stewart et al. 2015). In addition, the spectrally heterogeneous LMCs may also contribute to color vision together with the spectral-opponent photoreceptors.

### Lamina monopolar cells (LMCs) contribute to spectral processing

Does our new understanding of the spectral opponency of LMCs and photoreceptors allow us to better explain the wavelength discrimination ability of *Papilio*? Koshitaka et al. (2008) measured wavelength discrimination in trained *Papilio* and concluded using the receptor noise-limited color-opponent model that their color vision is tetrachromatic, based on the UV, B, G, and R receptors in the retina (Vorobyev and Osorio 1998).

**Fig. 12:**
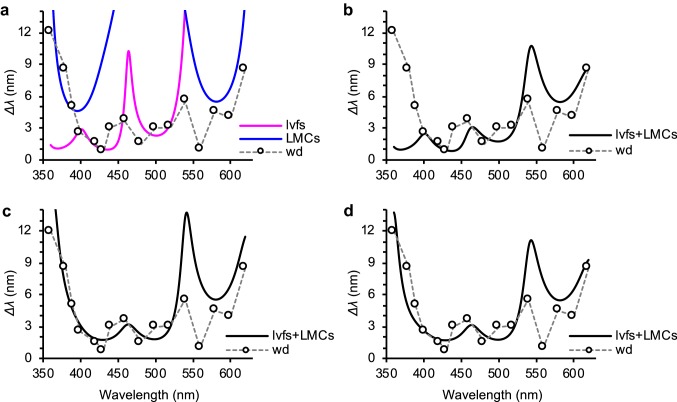
Comparison of modeled and behaviorally observed wavelength discrimination. Dotted and solid lines, respectively, indicate the previous behavioral data of wavelength discrimination (wd) of *Papilio* (Koshitaka et al. 2008) and predictions of our model. **a** Wavelength discrimination threshold calculated with all spectral classes of lvfs (magenta line; i.e., UV, violet, and blue lvfs) or all spectral types of LMCs (blue line; i.e., type I, II, and III LMCs). **b** All spectral classes of lvfs and all spectral types of LMCs combined. **c** As **b**, but with the V receptor and type II LMC excluded. **d** Like **b**, but with only the V receptor excluded

Here, we used the new set of inputs to calculate wavelength discrimination functions that can be compared to the previous behavioral results. We assumed that each cartridge contains two lvfs and four LMCs of a known spectral type (Matsushita et al., in preparation), equally contributing to the presumptive color vision mechanism. Figure 12 shows the calculation results under four sets of assumptions. We first assumed that only lvfs (magenta line) or only LMCs (blue line) from all ommatidial types participate in wavelength discrimination (Fig. 12a). The curve based solely on lvfs deviates considerably from the behavioral curve above 540 nm, while the curve based solely on LMCs somewhat coincides with the behavioral curve at the extremes of the spectrum, but strongly deviates in the middle. The calculated result exhibited serious discrepancy with the behavioral curve in the UV region when we assumed that all lvfs and all LMCs participate in color vision (Fig. 12b). As our previous study (Koshitaka et al. 2008) proposed that type II ommatidia are not involved in color vision, we removed all inputs from type II cartridges, which slightly improved the fit (Fig. 12c). However, reintroducing the type II LMC improves the fit further (Fig. 12d). The curve shown in Fig. 12d fits better than our previous photoreceptor-based calculations in short-wavelength regions, but not as well in long-wavelength regions (Koshitaka et al. 2008). This suggests that our understanding of the LMCs’ response properties is incomplete at this stage.

Nevertheless, our modeling indicates that the behavioral results in the short- and middle-wavelength range can be well explained by the assumption that chromatic information is conveyed by the spectrally opponent lvfs. The behavioral results in the long-wavelength range are reasonably well explained by the action of the spectrally heterogeneous LMCs. We propose that LMCs feed signals to the color vision system in parallel with the motion detection system (Stewart et al. 2015). Presumably, a better fit in the long-wavelength region could be obtained when we learn more about the anatomy and physiology of LMCs. Our finding of spectral-opponent LMCs (Fig. 5) already suggests that additional LMC classes with more diverse spectral properties could exist in the chromatic processing pathway.

### Interphotoreceptor connections as the source of opponency

Photoreceptors are interconnected in the lamina cartridges in an ommatidial type-specific manner in *Papilio* (Takemura and Arikawa 2006). We have previously hypothesized that a histamine-gated chloride channel, PxHCLB, is involved in these connections (Akashi et al. 2018; Chen et al. 2019). The present results clearly indicate that the interphotoreceptor connections are indeed inhibitory synapses that utilize chloride currents, providing further support for this account.

The inhibitory synapses between photoreceptors with different spectral sensitivities give rise to spectral opponency (Fig. 11), which has also been demonstrated in the *Drosophila* medulla where lvf pairs have mutual connections (Schnaitmann et al. 2018). As such synapses are absent from the fly lamina (Rivera-Alba et al. 2011), photoreceptor spectral opponency at the lamina has thus far only been observed in *Papilio*, though the feature may be common among butterflies. Only around a quarter of photoreceptors we recorded in the lamina showed spectral opponency. This variability is probably because not all photoreceptors are connected to each other; rather, the connection pattern is ommatidial type-specific (Takemura and Arikawa 2006). Also, the effect of synapses seems to be cumulative along the photoreceptor axons: The more proximal the recording site, the more prominent the hyperpolarization appears to be. Consistent with this account, all lvfs in the medulla showed even larger hyperpolarizing responses to light (Fig. 11).

The existence of spectral opponency at the photoreceptor level raises the question of whether and how it could enhance chromatic information processing. Photoreceptor spectral sensitivities are primarily determined by the absorption spectra of their visual pigments. The absorption spectra are often quite broad, which is beneficial for detecting light in general but detrimental to acute color vision. Photoreceptor spectral sensitivity is effectively broadened by the logarithmic amplification of the transduction cascade: For instance, a blue receptor whose visual pigment barely absorbs red light might still produce a physiologically meaningful response to red stimuli. However, this broadening is suppressed by spectral opponency. In butterflies, spectral tuning happens even in the retina, via a number of optical filtering effects (Stavenga 2002). Synaptic interactions further sharpen the spectral sensitivities in the lamina, as shown in Fig. 10; thus, spectral tuning is happening at multiple levels. We cannot determine at present which tuning mechanism—optical or neural—is evolutionarily older. Comparative anatomical studies of the retina and lamina could provide some insight into this question. Interestingly, some of the spectral sensitivities of *Papilio*’s spectral-opponent photoreceptors (Fig. 10) resemble those reported in higher-order color-opponent interneurons in honey bees and bumble bees (Kien and Menzel 1977; Paulk et al. 2009). It is notable that this complex processing occurs at such an early stage of the butterfly visual system.

We observed the modification of photoreceptor polarization sensitivities in the lamina (Fig. 9), which can also be explained by interphotoreceptor antagonistic interactions. Similar polarization opponency has been reported in flies (Hardie 1984; Weir et al. 2016). The fly lvfs R7 and R8 in the dorsal rim area are necessary and sufficient for polarotaxis (Wernet et al. 2012). These two photoreceptors have orthogonal polarization sensitivities and exhibit polarization opponency at their medulla terminals, likely due to mutual inhibition in the medulla (Weir et al. 2016). The antagonistic interaction between photoreceptors serves to enhance contrast not only in spectral but also in polarization signals.

It still remains to be understood whether and how the sharpened spectral sensitivities of photoreceptors and broad but heterogeneous spectral sensitivities of LMCs contribute to *Papilio*’s acute color vision. In *Drosophila*, R7 and R8 photoreceptors are presynaptic to the Dm8 amacrine neurons and a subset of medulla projection neurons, which appear to be involved in color learning (Melnattur et al. 2014). Identification of the counterparts of these neurons in the *Papilio* medulla would improve our understanding of the mechanism underlying the best color vision system known in the animal kingdom.
